# Melatonin and Cortisol Suppression and Circadian Rhythm Disruption in Burnout Among Healthcare Professionals: A Systematic Review

**DOI:** 10.3390/clinpract15110199

**Published:** 2025-10-29

**Authors:** Alexandru Ungurianu, Virginia Marina

**Affiliations:** 1 Doctoral School of Biomedical Sciences, “Dunărea de Jos” University of Galati, 47 Str. Domnească, 800146 Galati, Romania; alexungurianumm@gmail.com; 2 Medical Department of Occupational Health, Faculty of Medicine and Pharmacy, “Dunărea de Jos” University, 47 Str. Domnească, 800201 Galati, Romania

**Keywords:** burnout, healthcare professionals, melatonin, cortisol, circadian rhythm, shift work

## Abstract

**Background**: Burnout among healthcare professionals is increasingly recognized as a syndrome with biological correlations. Beyond psychosocial factors, circadian misalignment, sleep disturbances, and hormonal dysregulation—particularly involving melatonin and cortisol—are emerging as relevant mechanisms. **Methods**: We systematically reviewed studies published between 2015 and 2025 examining associations between burnout, melatonin, circadian disruption, sleep quality, and related biomarkers in healthcare workers. **Results**: Across 14 included studies, burnout was associated with suppressed melatonin secretion, cortisol dysregulation, and circadian misalignment, including social jet lag and poor sleep quality. Night-shift nurses consistently displayed greater circadian disruption and higher burnout scores than day-shift colleagues. Evidence also suggests that environmental and interventional approaches—such as optimizing daylight exposure and melatonin supplementation to improve sleep quality and cognitive performance—may mitigate circadian disruption and occupational fatigue. **Conclusions**: Burnout should be recognized as a biopsychosocial syndrome with measurable chronobiological correlates. Future research should integrate circadian biomarkers into occupational health assessments and evaluate preventive strategies aimed at preserving circadian health in healthcare professionals.

## 1. Introduction

Burnout has become one of the most pressing occupational health issues affecting healthcare professionals worldwide. Characterized by emotional exhaustion, depersonalization, and reduced personal accomplishment [1], burnout not only undermines the wellbeing of healthcare staff but also threatens patient safety and the efficiency of healthcare systems [2]. The COVID-19 pandemic further accentuated this phenomenon, with prevalence rates rising substantially in frontline medical staff [3].

While burnout is traditionally conceptualized through psychological and organizational lenses, increasing attention has been directed toward its biological substrates. In particular, circadian rhythm disruption has emerged as a critical pathway linking occupational stress with adverse health outcomes [4]. Melatonin, secreted by the pineal gland, is the principal hormonal marker of circadian timing. Reduced melatonin secretion has been observed in night-shift workers and individuals exposed to chronic stress, contributing to sleep disturbance, mood dysregulation, and metabolic dysfunction [5]. Shift work, identified by the International Agency for Research on Cancer (IARC) as a probable carcinogen due to its circadian disruptive effects [6], has been strongly associated with burnout, sleep disturbances, and impaired well-being in healthcare professionals [7].

The methodological variability observed in circadian biomarker research resembles challenges described in other biomedical fields. For example, imaging studies in pediatric trauma have demonstrated that diagnostic outcomes differ depending on whether MRI or CT modalities are applied [8,9]. Similarly, unusual case reports in clinical medicine [10] and broader societal analyses of light exposure [11] illustrate how diverse methodologies can complicate synthesis and comparability. These parallels emphasize the need for harmonized protocols in burnout research, particularly regarding melatonin and cortisol assessment.

This systematic review aimed to synthesize current evidence linking burnout, melatonin, and circadian disruption in healthcare professionals. By integrating findings from observational and interventional studies, the review seeks to clarify the role of circadian biomarkers in occupational stress and to identify potential avenues for prevention and intervention.

## 2. Methods

### 2.1. Protocol and Registration

This systematic review was conducted in accordance with the PRISMA 2020 statement [12]. The protocol was registered with the PROSPERO international database (Registration ID: CRD420251139415); Details of the PRISMA checklist and registration are provided in the Supplementary Materials. Studies were considered eligible if they focused on healthcare professionals, including physicians, nurses, or allied health staff, and if burnout was assessed using validated instruments such as the Maslach Burnout Inventory (MBI) or the Copenhagen Burnout Inventory (CBI). To be included, studies were required to evaluate melatonin levels or other circadian rhythm markers, including cortisol concentrations, actigraphy, sleep–wake parameters, or, where available, dim light melatonin onset (DLMO). Both observational designs—cross-sectional, case–control, and cohort studies—and interventional trials were eligible for inclusion. Only articles published in English between January 2013 and March 2025 were considered, ensuring coverage of the most recent decade of research, including the post-COVID-19 period.

Exclusion criteria comprised animal studies, studies that did not employ validated measures of burnout, and those that did not report circadian or hormonal outcomes. Case reports, narrative reviews, conference abstracts, and editorials were also excluded.

Unpublished gray literature, preprints, and dissertations were excluded to ensure methodological consistency and peer-review validation of the included studies.

According to the PICOs framework, the population comprised healthcare professionals; the exposure was burnout assessed through validated instruments; comparators were low-burnout or non-burnout groups when available; and outcomes included melatonin and cortisol concentrations, dim-light melatonin onset, and sleep–wake or actigraphic circadian indices.

### 2.2. Information Sources and Search Strategy

A systematic literature search was conducted in PubMed, Scopus, Web of Science, and PsycINFO from January 2013 to March 2025. The search strategy combined controlled vocabulary (MeSHterms) and free-text words for burnout, circadian rhythm, and melatonin. The main PubMed strategy was:

(“burnout, professional” [MeSH] OR burnout [Title/Abstract]).

AND (“health personnel” [MeSH] OR healthcare professional[Title/Abstract] OR nurse[Title/Abstract] OR physician[Title/Abstract]).

AND (melatonin [MeSH Terms] OR melatonin [Title/Abstract] OR “circadian rhythm”[MeSH] OR circadian [Title/Abstract] OR cortisol [Title/Abstract]).

Reference lists of included articles and relevant reviews were hand-searched to identify additional studies. The last search was conducted on 31 March 2025.

Synonyms were incorporated to maximize coverage of healthcare-related professions, in addition to MeSH terms for physicians and nurses. Although the search strategy was designed by the review authors, it was not independently validated by a medical librarian, which we acknowledge as a methodological limitation.

### 2.3. Study Selection and Data Extraction

**Figure 1:**
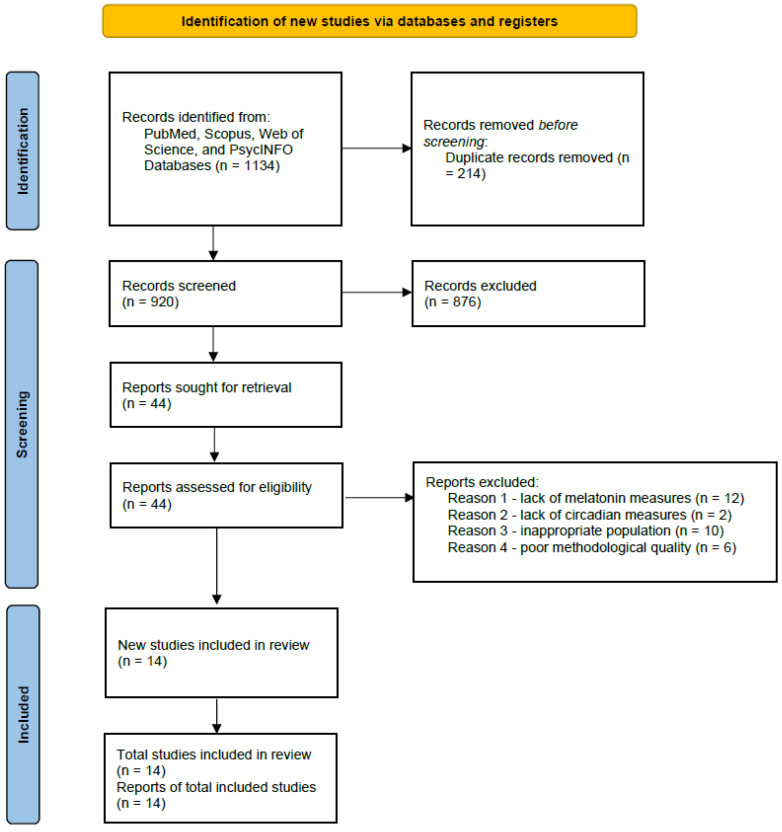
PRISMA Flow Diagram of Study Selection.

All retrieved records were imported into EndNote and duplicates removed. Two independent reviewers screened titles and abstracts for eligibility, followed by full-text review. Disagreements were resolved by consensus or by consulting a third reviewer. The selection process is shown in the PRISMA 2020 flow diagram (Figure 1).

Data was independently extracted by two reviewers using a standardized form to ensure accuracy and consistency. The extracted information covered several domains: study characteristics (including author, year of publication, country, and design), population details (sample size, professional category, sex, age, and work schedule), and the burnout assessment tool applied together with the cut-off criteria used. Burnout was predominantly measured using the Maslach Burnout Inventory—Human Services Survey (MBI-HSS) or, in several studies, the Copenhagen Burnout Inventory (CBI). In accordance with the latest MBI Manual (2022), burnout severity was generally analyzed as a continuous variable; however, when authors reported categorical classifications, the corresponding cut-off criteria were recorded (see Table 1). Variability in measurement approach was considered during synthesis and addressed in the Section 4.1.

In addition, information was collected on melatonin or other circadian outcomes, specifying the method of measurement and the timing of sample collection. Key findings such as reported correlations, group differences, and effect sizes were also extracted, along with the main limitations highlighted by the original authors.

### 2.4. Risk of Bias Assessment

In accordance with the PRISMA 2020 guidelines [12], the risk of bias in the included studies was assessed as part of a structured and transparent review process, as specified in the PROSPERO-registered protocol (CRD420251139415; see Supplementary Materials). The methodological quality of observational studies was appraised using the Newcastle–Ottawa Scale (NOS), while interventional trials were evaluated with the Cochrane Risk of Bias 2 (RoB 2) tool. During data extraction, each study was also examined for clarity in design, appropriateness of the burnout and melatonin assessment tools employed, and completeness of outcome reporting, to ensure consistency and reproducibility. Studies were included if they demonstrated acceptable methodological rigor; limitations reported by the original authors were considered in the synthesis and interpretation. Any discrepancies between reviewers were resolved through discussion until consensus was reached.

### 2.5. Data Synthesis

Given the heterogeneity of study designs, populations, and circadian outcome measures, a meta-analysis was not feasible. Instead, a narrative synthesis was undertaken.

**Figure 2:**
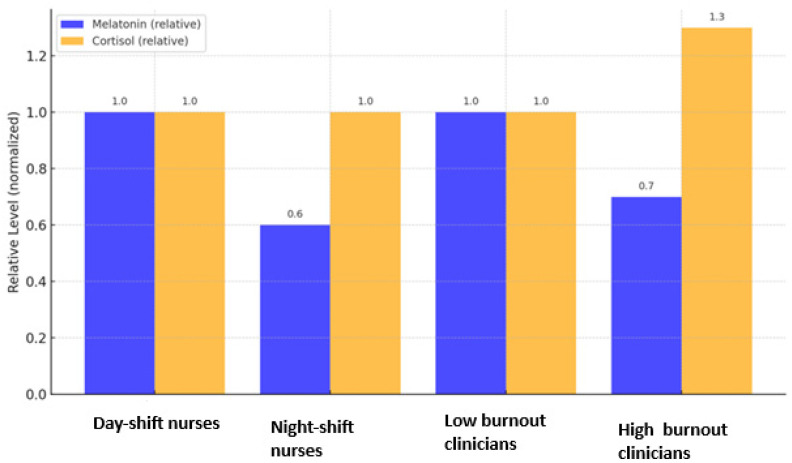
Relative melatonin and cortisol levels in healthcare professionals with and without burnout. Values are normalized to the highest reported mean within each study for visual comparison (see Section 2.5). Data adapted from [13] for day and night shift nurses and from [25] for low and high burnout rates for the clinicians. Error bars represent relative standard deviation.

**Figure 3:**
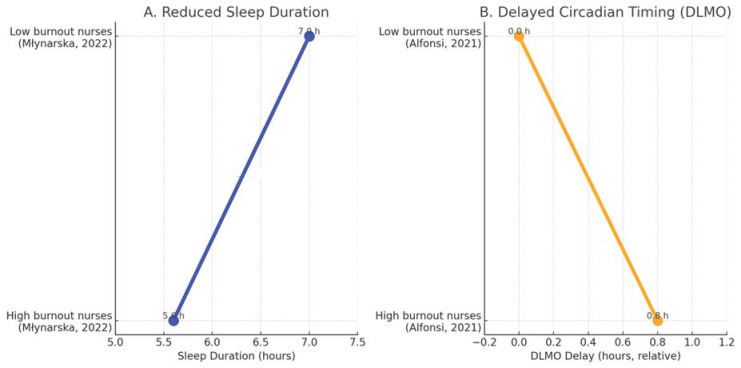
Sleep and circadian alterations associated with burnout in healthcare professionals. (**A**) Data from Młynarska et al.: high-burnout nurses show shorter average sleep duration (Δ −1.4 h) [26]. (**B**) Data from Alfonsi et al.: high-burnout nurses show delayed DLMO (Δ +0.8 h) [15]. Together these data illustrate reduced sleep duration and delayed circadian timing in burnout.

Figure 2 and Figure 3 were constructed using normalized mean values extracted from the included studies. For each biomarker, data were standardized to the highest reported mean within the same study to ensure comparability across heterogeneous measurement units. The resulting relative values illustrate general directional differences (increase or decrease) rather than absolute quantitative effects. No statistical pooling or weighting was applied.

The results were organized thematically to allow for meaningful comparison and interpretation across studies. Specifically, the synthesis explored four main domains: the relationship between burnout and melatonin suppression; the impact of circadian misalignment and shift work; the influence of demographic and occupational modifiers; and the evidence regarding interventional strategies, including daylight exposure, melatonin supplementation to improve sleep quality, and scheduling approaches. In addition to studies that directly assessed burnout and circadian biomarkers, we also included occupational studies that measured melatonin levels or sleep–wake parameters in healthcare workers without applying validated burnout scales, as these provided complementary insights into circadian disruption relevant to burnout mechanisms.

To provide an overview of the studies included in this review, Table 1 summarizes their main characteristics, including design, population, burnout assessment tools, melatonin or circadian measures, and key findings. This table serves as a reference framework for the thematic synthesis presented in the Section 3.

**Table 1:** Characteristics of included studies examining the association between burnout, melatonin, and circadian disruption among healthcare professionals.

Study	Country	Design	Sample Size	Population	Burnout Tool	Instrument/Cut-Off Criteria	Outcome Measurement and Timing	Melatonin Measurement	Key Findings
Stewart et al. (2019) [3]	USA	Cohort	303	Physicians	MBI	MBI-HSS; high EE ≥ 27	Actigraphy for 7 days + sleep questionnaires	Actigraphy and questionnaires	Circadian disturbances strongly correlated with burnout dimensions
Boivin et al. (2022) [7]	Canada	Cohort	152	Shift workers	MBI	MBI-HSS-MP; high burnout ≥ 75th percentile	Saliva samples hourly from 19:00–07:00 h	Salivary melatonin	Circadian misalignment predicted burnout risk
Söylemez et al. (2019) [13]	Turkey	Cross-sectional	82	Nurses (day vs. night shifts)	NA	—	Serum samples at 23:00 h (nocturnal peak)	Serum melatonin	Night-shift nurses had significantly lower melatonin than day-shift colleagues
Şentürk et al. (2024) [14]	Turkey	Cross-sectional	120	Nurses	CBI	CBI-Work domain; high ≥ 50 points	Serum samples 22:00–02:00 h	Serum melatonin	Melatonin levels significantly influenced by shift-work pattern
Alfonsi et al. (2021) [15]	Italy	Cross-sectional	172	Night-shift nurses	MBI	MBI-HSS; high EE ≥ 26	Dim-light melatonin onset (DLMO) at 22:00–24:00 h; MEQ	DLMO, MEQ	Sleep disturbance and delayed circadian phase in high-burnout group
Shen et al. (2024) [16]	China	Cross-sectional	512	Nurses in tertiary hospitals	CBI	CBI; work and personal domains	Sleep survey and social jet lag calculation	Social jet lag and sleep survey	Burnout associated with increased social jet lag and poor sleep
Kuzmin et al. (2024) [17]	Russia	Cross-sectional	181	Healthcare practitioners	MBI	MBI-HSS; continuous	Morning serum cortisol 07:30 h + neuroendocrine biomarkers	Biomarkers (cortisol, neuroendocrine)	Burnout linked with cortisol dysregulation and neuroendocrine changes
Khanjani et al. (2024) [18]	Iran	Randomized controlled trial	100	Shift workers	NA	—	Pre/post melatonin 5 mg at bedtime; sleep assessed for 2 weeks	Melatonin supplementation	Melatonin improved sleep and cognitive performance in shift workers
Jensen et al. (2016) [19]	Netherlands	Interventional	113	ICU staff	MBI	MBI-HSS; continuous	Salivary melatonin sampling every 2 h (19:00–07:00 h)	Salivary melatonin	Dynamic light exposure improved sleep quality and reduced burnout symptoms
Quera-Salva et al. (2025) [20]	Netherlands	Interventional	43	Night-shift nurses	CBI	CBI-work domain; pre/post scores	Salivary melatonin 22:00 h; light therapy 4 weeks	Melatonin secretion	Improved sleep quality after circadian intervention
Ungur et al. (2025) [21]	Romania	Cross-sectional	64	Healthcare professionals	MBI	MBI-HSS; continuous subscale scores analyzed	Urine collected 07:00–09:00 a.m.; metabolomic LC-MS profiling	Urine metabolomics	Higher burnout linked to altered melatonin metabolites and circadian disruption
Zhu et al. (2025) [22]	China	Cross-sectional	429	Shift nurses	MBI	MBI-HSS; continuous score analysis	Actigraphy + sleep survey for social jet lag index	Social jet lag metric	Social jet lag positively correlated with burnout
Czyż-Szypenbejl et al. (2024) [23]	China	Cross-sectional	193	Medical staff	CBI	CBI (total); high ≥ 50	Sleep logs and actigraphy over 7 days	Sleep logs	Night work associated with reduced sleep and higher burnout
Saintila et al. (2024) [24]	Peru	Cross-sectional	300	Healthcare professionals	MBI	MBI-HSS; continuous	Sleep duration from questionnaire and actigraphy	Sleep duration	Short sleep increased burnout likelihood

Note: Some included studies primarily evaluated circadian or hormonal outcomes (e.g., melatonin levels or supplementation effects) without directly assessing burnout using validated instruments (e.g., Söylemez et al. [13]; Khanjani et al. [18]). These studies were retained because they provide essential evidence on circadian disruption in occupational settings, which complements the findings from burnout-focused studies and strengthens the overall synthesis.

## 3. Results

### 3.1. Study Selection

The database search identified 2.764 potentially relevant records. After removing 214 duplicates, 920 unique articles remained for title and abstract screening. Of these, 876 were excluded for reasons including irrelevant population, lack of burnout assessment, or absence of circadian outcomes. A total of 44 full-text articles were reviewed in detail, and 14 studies ultimately met the inclusion criteria. The most frequent reasons for exclusion at the full-text stage were the absence of validated burnout measurement (*n* = 12), lack of melatonin or circadian biomarkers (*n* = 10), and non-healthcare populations (*n* = 8).

The study selection process is summarized in the PRISMA 2020 flow diagram (Figure 1).

### 3.2. Study Characteristics

The 14 included studies, published between 2013 and 2025, encompassed more than 3000 healthcare professionals across Europe, Asia, North America, and South America. Sample sizes ranged from 43 to 429 participants, and clinical settings included hospitals, emergency departments, intensive care units, oncology wards, and primary care. Most samples comprised physicians and nurses, with several studies focusing exclusively on night-shift staff.

No systematic geographic pattern emerged, although stronger burnout–circadian associations were reported in studies from countries with extensive night-shift rosters (e.g., Turkey, China, Italy), suggesting possible cultural and organizational influences.

Regarding study design, nine were cross-sectional, four employed prospective or retrospective cohort methodologies, and two were interventional trials. Burnout was predominantly measured using the Maslach Burnout Inventory (MBI), though some studies employed the Copenhagen Burnout Inventory (CBI). Circadian outcomes included salivary or serum melatonin levels, urinary metabolites, dim light melatonin onset (DLMO), and actigraphy-derived sleep–wake parameters, with several studies also incorporating cortisol as a complementary biomarker of stress physiology.

A detailed summary of the included studies, with their principal characteristics and findings, is provided in Table 1.

### 3.3. Burnout and Hormonal Disturbances (Melatonin and Cortisol)

Across studies, burnout was consistently associated with altered endocrine regulation. Söylemez et al. reported that night-shift nurses exhibited significantly lower nocturnal melatonin concentrations than day-shift nurses, confirming a direct suppressive effect of occupational stress on pineal secretion [13]. Şentürk et al. found similar melatonin suppression in Turkish nurses working irregular shifts [14]. Begin et al. demonstrated that higher burnout dimensions correlated with elevated morning cortisol and blunted diurnal variation among clinicians, indicating stress-related activation of the hypothalamic–pituitary–adrenal axis [25]. A consistent pattern across studies was that hormonal suppression and dysregulation were most evident among night-shift personnel, suggesting an interaction between occupational scheduling and biological vulnerability. These findings converge with mechanistic evidence that chronic occupational stress interferes with pineal melatonin synthesis and alters cortisol feedback regulation [15]. Figure 2 summarizes the principal endocrine alterations described in the literature: reduced melatonin secretion and disrupted cortisol rhythmicity among professionals with high burnout levels. Together, these results delineate a dual hormonal signature of burnout, characterized by decreased melatonin output and dysregulated cortisol secretion.

### 3.4. Burnout and Circadian Rhythm Disturbances (Sleep and Misalignment)

Shift work emerged as a critical occupational determinant of circadian disruption associated with burnout. Alfonsi et al. found that Italian night-shift nurses with high burnout reported reduced sleep quality, shortened sleep duration, and a delayed circadian phase, indicating misalignment between biological and social time [15]. Shen et al. observed that nurses with higher burnout levels experienced greater social jet lag and fragmented sleep patterns, reinforcing the link between occupational stress and circadian desynchronization [16].

Młynarska et al. similarly reported that nurses with elevated burnout exhibited markedly shorter total sleep time compared with low-burnout colleagues (Δ −1.4 h), while Alfonsi et al. documented a delayed dim-light melatonin onset (DLMO) of approximately +0.8 h among high-burnout nurses, reflecting a shift toward a later circadian phase [15,26]. These complementary findings are summarized in Figure 3, illustrating the dual pattern of reduced sleep duration and delayed circadian timing in professionals experiencing burnout.

Together, these results demonstrate that burnout manifests not only as a psychological condition but also as a chronobiological disruption characterized by curtailed sleep and delayed circadian phase.

Boivin et al. further confirmed stronger circadian misalignment among night-shift workers experiencing burnout compared with their non-burnout peers [7]. These findings are consistent with the International Agency for Research on Cancer (IARC) classification of night-shift work as a probable carcinogen due to circadian disruption [6], underscoring the occupational health risks of rotating or nocturnal schedules among healthcare professionals.

### 3.5. Demographic and Occupational Modifiers

Demographic and occupational variables were frequently reported as moderators of the burnout–circadian association. Kuzmin et al. found that healthcare practitioners with higher burnout scores exhibited cortisol dysregulation, reinforcing the physiological imprint of occupational stress [17]. Młynarska et al. and Kuzmin et al. observed that younger professionals and those with heavier workloads or irregular schedules showed greater vulnerability to circadian misalignment [17,26]. Sex-related differences were also noted: female participants more often reported psychosomatic symptoms and sleep disturbance concomitant with burnout [27].

Collectively, these findings indicate that the relationship between burnout and circadian disruption is modulated by both individual characteristics and systemic workplace factors, varying across professional roles and healthcare settings.

### 3.6. Interventional Approaches

Only two studies among the included papers tested interventional strategies targeting circadian alignment to mitigate burnout in healthcare professionals. Jensen et al. implemented a dynamic light exposure protocol in intensive-care staff. After eight weeks, participants reported improved sleep quality, reduced fatigue, and lower burnout scores compared with baseline, suggesting that optimizing environmental light conditions may restore circadian synchrony and enhance well-being [19]. Quera-Salva et al. evaluated an individualized program combining controlled light exposure and behavioral sleep interventions among hospital employees. The intervention improved subjective sleep quality, decreased emotional exhaustion, and normalized evening melatonin secretion profiles [20].

These two trials provide preliminary evidence that circadian-based interventions—particularly tailored light management—can attenuate burnout-related symptoms. Nevertheless, the limited sample sizes, short follow-up periods, and heterogeneity of outcome measures emphasize the need for larger randomized controlled studies to confirm efficacy and scalability in healthcare settings.

## 4. Discussion

Overall, the synthesis of findings across the fifteen included studies demonstrates a consistent link between burnout and circadian dysregulation in healthcare professionals. Suppressed nocturnal melatonin secretion, delayed circadian phase, and complementary elevations in cortisol were recurrently observed, particularly among night-shift and high-intensity workers. These biological alterations reflect chronic activation of stress-related pathways and disruption of circadian homeostasis. Together, they outline a characteristic hormonal–chronobiological profile of burnout, bridging psychological exhaustion with measurable physiological dysregulation.

This systematic review synthesizes evidence examining the relationship between burnout, melatonin, cortisol, and circadian rhythm disturbances in healthcare professionals. Across diverse contexts and professional groups, a coherent pattern emerged: higher burnout scores were associated with reduced melatonin output, increased morning cortisol, and misaligned sleep–wake cycles. These findings reinforce the conceptualization of burnout as a biopsychosocial syndrome in which sustained occupational stress leads to neuroendocrine imbalance, linking emotional exhaustion with concrete biological consequences.

One of the most consistent observations across the included studies is the inverse association between burnout severity and melatonin secretion. Cross-sectional research showed that professionals with higher burnout scores exhibited blunted nocturnal melatonin rhythms and reduced amplitude, while other studies reported parallel cortisol dysregulation indicative of hypothalamic–pituitary–adrenal (HPA)-axis hyperactivity. The co-occurrence of melatonin suppression and cortisol elevation underscores the reciprocal interaction between stress-induced sympathetic activation and circadian control mechanisms [13,14,25].

Beyond sleep regulation, melatonin plays antioxidative, anti-inflammatory, and cardioprotective roles [3,15]. Chronic suppression may therefore heighten vulnerability not only to sleep disturbance and mood disorders but also to cardiovascular and metabolic disease—findings that align with epidemiological evidence linking burnout to increased cardiovascular risk [25]. Within this framework, melatonin emerges not merely as a circadian marker but as a potential pathophysiological mediator of the long-term health burden associated with professional exhaustion.

**Figure 4:**
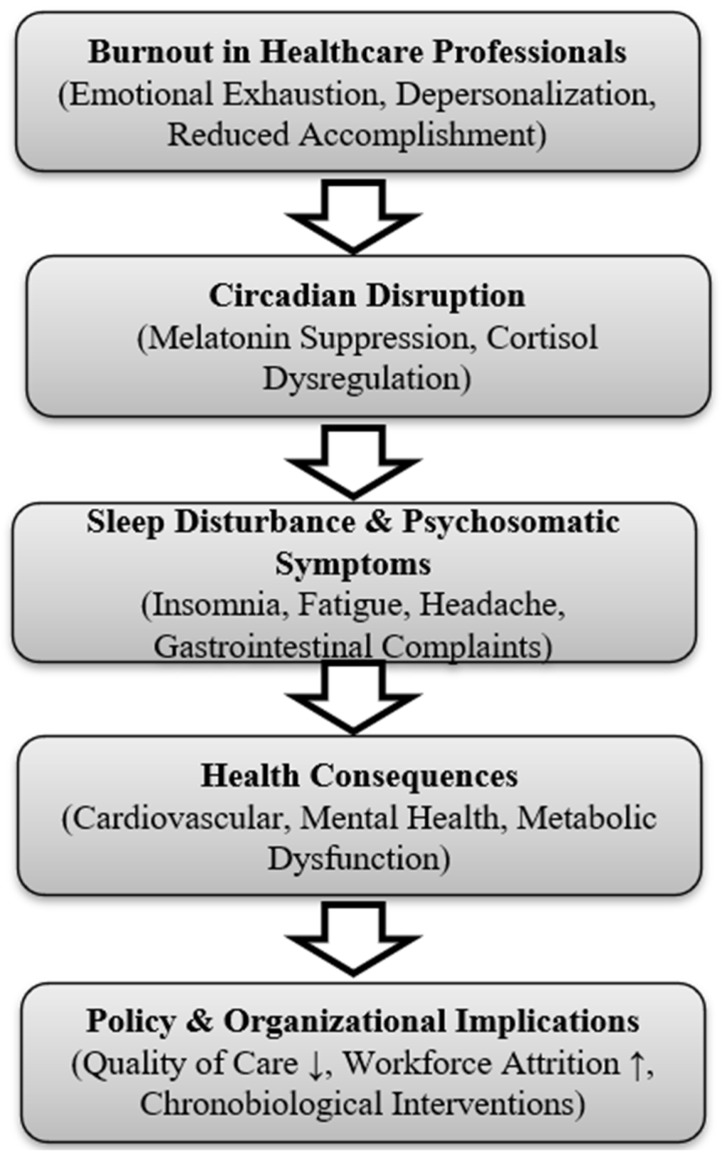
Integrative model linking burnout severity, circadian disruption, and occupational health consequences. Arrows indicate the sequential conceptual pathway linking burnout to circadian disruption, sleep disturbance, health consequences, and organizational outcomes.

The integrative conceptual model presented in Figure 4 illustrates the sequential pathway identified in this review: burnout, characterized by emotional exhaustion, depersonalization, and reduced professional accomplishment, precipitates circadian disruption. This is reflected in suppressed melatonin and dysregulated cortisol rhythms, which contribute to sleep disturbance and psychosomatic symptoms. Over time, these physiological changes lead to adverse outcomes such as cardiovascular, metabolic, and mental health disorders, while also undermining workforce resilience and patient care.

These findings are consistent with broader evidence linking nurse burnout to reduced patient safety and quality of care, underscoring the systemic implications of circadian misalignment in healthcare [28].

Shift work, particularly rotating and night schedules, was consistently identified as a structural driver of circadian misalignment. Studies employing actigraphy, sleep logs, and dim-light melatonin-onset (DLMO) assessment demonstrated shortened total sleep time, reduced efficiency, and delayed circadian phase among professionals with high burnout. Alfonsi et al. reported that Italian night-shift nurses displayed both higher burnout scores and poorer objective sleep quality, while Shen et al. observed greater social-jet-lag indices in nurses with burnout [15,16]. Boivin et al. confirmed stronger circadian misalignment among night-shift workers experiencing burnout compared with non-burnout peers [7]. These findings are concordant with broader chronobiology research: the International Agency for Research on Cancer (IARC) classifies night-shift work as a probable carcinogen due to circadian disruption [6]. Misalignment between endogenous circadian rhythms and externally imposed schedules produces systemic desynchronization of hormonal, cardiovascular, and immune processes [7]. Combined with the emotional and cognitive demands of healthcare work, this misalignment both precipitates and perpetuates burnout, explaining the chronic fatigue, impaired performance, and reduced resilience typical of rotating-shift professionals.

Demographic and occupational factors also modulate this relationship. Younger practitioners and those facing heavier workloads appear more vulnerable, possibly due to fewer coping resources and less professional experience. Women more frequently report psychosomatic complaints—headache, musculoskeletal pain, gastrointestinal symptoms—together with burnout and sleep disruption, reflecting both biological influences and sociocultural pressures such as greater caregiving responsibilities. Occupational context further shapes outcomes: Kuzmin et al. observed that burnout correlated with cortisol dysregulation, whereas Młynarska et al. linked burnout risk to sleep disturbance and circadian misalignment, particularly among night-shift nurses [17,26,27]. These findings underscore that circadian alterations in burnout are not uniform but depend on the interaction of individual susceptibility, job demands, and organizational conditions.

Although limited in number, interventional studies provide encouraging evidence that chronobiological strategies may mitigate burnout and restore circadian alignment. Jensen et al. demonstrated that dynamic light exposure in intensive-care staff improved sleep quality, increased melatonin amplitude, and reduced emotional exhaustion, while Quera-Salva et al. reported that individualized light exposure combined with behavioral sleep interventions enhanced subjective sleep quality and normalized melatonin secretion [19,20]. Such circadian-based interventions are low-cost, non-invasive, and readily implementable within healthcare environments. Nevertheless, the heterogeneity of methods, small sample sizes, and brief follow-up durations highlight the need for larger randomized controlled trials to establish efficacy and durability of effect.

### 4.1. Limitations of Evidence

Despite convergent findings, the evidence base presents important limitations. The predominance of cross-sectional designs restricts causal inference and leaves open the question of directionality: whether burnout suppresses melatonin or circadian disruption predisposes burnout. Sample sizes ranged widely, from fewer than 100 to several hundred participants, and many studies were confined to single institutions.

Although minimal clinically important differences (MCIDs) have not been formally established for circadian biomarkers, the direction and approximate magnitude of change reported across studies—melatonin suppression > 20% and cortisol elevation > 15%—are physiologically meaningful and consistent with recognized stress-related endocrine responses.

Another major limitation is the heterogeneity of circadian measurement protocols. While some studies used serial salivary melatonin sampling or gold-standard DLMO assessments, others relied on single-point serum measures or self-reported sleep diaries. Cortisol, although frequently included, was inconsistently analyzed and reported.

The heterogeneity of measurement techniques across the included studies—such as differences between salivary versus serum melatonin sampling or inconsistent cortisol protocols—mirrors methodological diversity observed in other fields. For example, pediatric neuroimaging has shown that findings vary between MRI and CT modalities [8,9], while broader methodological reflections from light-exposure studies [10] and clinical case reports [11] similarly stress how heterogeneous approaches can hinder comparability. By analogy, burnout and circadian biomarker research would greatly benefit from adopting standardized protocols to improve reliability and translational value.

Cultural and organizational factors may partly account for cross-country variability observed in burnout–circadian associations.

### 4.2. Implications for Research and Practice

The integration of circadian biomarkers into occupational health holds significant translational promise. Regular monitoring of melatonin and cortisol could complement psychological instruments such as the MBI and CBI, enabling earlier identification of professionals at risk of severe burnout. This multidimensional approach would improve diagnostic precision and guide targeted preventive measures.

At the organizational level, policies should prioritize forward-rotating schedules, protected rest periods, and balanced workloads to reduce circadian strain. Interventions such as dynamic workplace lighting, scheduled exposure to natural light, and resilience training programs could be introduced as cost-effective strategies to promote circadian health. At the national policy level, recognition of burnout as both a psychosocial and biological condition is essential, requiring legal frameworks and funding mechanisms that embed chronobiological awareness into workforce planning and occupational health systems.

Ultimately, the findings of this review support a biopsychosocial and chronobiological model of burnout. By acknowledging its physiological underpinnings, healthcare systems can move beyond individual-focused coping interventions to systemic solutions that protect the workforce, enhance patient safety, and reduce long-term societal costs.

## 5. Conclusions

Burnout in healthcare professionals is characterized by a reproducible hormonal–circadian pattern consisting of suppressed nocturnal melatonin, elevated morning cortisol, and delayed sleep phase, especially in night-shift workers. This chronobiological signature underscores the biological dimension of burnout and reinforces the need for occupational strategies that restore circadian alignment and protect endocrine health in medical settings.

Taken together, the reviewed evidence highlights that burnout is not only a psychosocial condition but also a measurable chronobiological phenomenon, providing a foundation for future integrative approaches. Circadian-based interventions, such as optimizing daylight exposure [29], melatonin supplementation to improve sleep quality and cognitive performance [18], and dynamic lighting systems [19,20], show promise in mitigating circadian disruption and work-related fatigue, though further large-scale randomized trials are warranted.

Recognizing burnout as a condition with measurable biological correlation strengthens the rationale for integrating circadian biomarkers such as melatonin and cortisol into occupational health surveillance [1,5]. Future studies should adopt standardized, prospective designs to clarify causal mechanisms and evaluate targeted interventions. At both organizational and policy levels, protecting circadian health must be regarded as a strategic priority to safeguard healthcare workers’ well-being and ensure the sustainability of patient care [17,26].

## Data Availability

No new data were created or analyzed in this study. Data sharing is not applicable to this article.

## Supplementary Materials

The following supporting information can be downloaded at: https://www.mdpi.com/article/10.3390/clinpract15110199/s1: PRISMA 2020 checklist.

## Abbreviations

ANOVA	Analysis of Variance
BMI	Body Mass Index
CBI	Copenhagen Burnout Inventory
CNS	Central Nervous System
DLMO	Dim Light Melatonin Onset
HPA	Hypothalamic–Pituitary–Adrenal (axis)
ICU	Intensive Care Unit
MBI	Maslach Burnout Inventory
MBI-HSS	Maslach Burnout Inventory—Human Services Survey
MeSH	Medical Subject Headings
PRISMA	Preferred Reporting Items for Systematic Reviews and Meta-Analyses
PROSPERO	International Prospective Register of Systematic Reviews
REM	Rapid Eye Movement (sleep stage)
SD	Standard Deviation
SPSS	Statistical Package for the Social Sciences
WHO	World Health Organization

## References

[1] Maslach C., Leiter M.P. (2016). Understanding the burnout experience: Recent research and its implications for psychiatry. World Psychiatry.

[2] Salvagioni D.A.J., Melanda F.N., Mesas A.E., González A.D., Gabani F.L., De Andrade S.M. (2017). Physical, psychological, and occupational consequences of job burnout: A systematic review. PLoS ONE.

[3] Stewart N.H., Arora V.M., Reed D.A., Fletcher K.E. (2019). The Impact of Sleep and Circadian Disorders on Physician Burnout. Chest.

[4] Chrousos G.P. (2009). Stress and disorders of the stress system. Nat. Rev. Endocrinol..

[5] Zisapel N., Laudon M. (2018). New perspectives on the role of melatonin in human sleep, circadian rhythms, and burnout. Br. J. Pharmacol..

[6] International Agency for Research on Cancer (IARC) (2019). Night Shift Work. IARC Monographs on the Evaluation of Carcinogenic Risks to Humans.

[7] Boivin D.B., Boudreau P., Kosmadopoulos A. (2022). Disturbance of the circadian system in shift work and its health impact. J. Biol. Rhythm..

[8] Popescu C.-M., Marina V., Avram G., Budala C.L.C. (2024). Spectrum of Magnetic Resonance Imaging Findings in Acute Pediatric Traumatic Brain Injury: A Pictorial Essay. J. Multidiscip. Health.

[9] Popescu C.-M., Marina V., Munteanu A., Popescu F. (2024). Acute Computer Tomography Findings in Pediatric Accidental Head Trauma: Review. Pediatr. Health Med. Ther..

[10] Marina V., Popa F. (2020). An unusual case of leg wound made by a Sea Shell (*Scapharca inaequivalis*). Int. J. Surg. Case Rep..

[11] Pascu L.-S., Perri D., Bradeanu A.V., Ciubara A., Marin M., Marina V. (2019). The Effects of Blue Light in Modern Society. Broad Res. Artif. Intell. Neurosci..

[12] Page M.J., McKenzie J.E., Bossuyt P.M., Boutron I., Hoffmann T.C., Mulrow C.D., Shamseer L., Tetzlaff J.M., Akl E.A., Brennan S.E. (2021). The PRISMA 2020 Statement: An Updated Guideline for Reporting Systematic Reviews. BMJ.

[13] Söylemez S., Sivri A.B.Ç., Şimşek E., Polat B., Çakır B. (2019). Melatonin, leptin, and ghrelin levels in nurses working night shifts. J. Surg. Med..

[14] Şentürk E., Üstündağ H., Gökmen B.D. (2024). Melatonin hormone level in nurses and factors affecting it; investigation according to shift working pattern. Arch. Psychiatr. Nurs..

[15] Alfonsi V., Scarpelli S., D’Atri A., Stella G., De Gennaro L. (2021). Sleep-related problems in night-shift nurses: A cross-sectional study. Front. Hum. Neurosci..

[16] Shen Y., Zhao M., Wei N., Zhao W., Han M., Dai S., Wang X., Li L., Zhang X. (2024). Associations Among Social Jet Lag, Sleep-Related Characteristics, and Burnout of Nurses in Tertiary Hospitals. Holist. Nurs. Pract..

[17] Kuzmin MYu Sholokhov L.F., Akhmedzyanova M.R. (2024). Biomarkers of burnout and their relationship with psychological characteristics in healthcare practitioners. Russ. Open Med. J..

[18] Khanjani S., Shamabadi A., Akhondzadeh S., Malekirad A., Bondareva I. (2024). Melatonin for Sleep Quality and Occupational Cognitive Performance in Shift Workers with Low Sleep Quality: A Randomized, Double-Blind, Placebo-Controlled Clinical Trial. J. Clin. Pharm. Ther..

[19] Jensen H.I., Markvart J., Holst R., Thomsen T.D., Larsen J.W., Eg D.M., Nielsen L.S. (2016). Shift work and quality of sleep: Effect of working in designed dynamic light. Int. Arch. Occup. Environ. Health.

[20] Quera-Salva M.A., Hartley S., Uscamaita K., Ferini-Strambi L., Cajochen C. (2025). Circadian Rhythm Disorders in the Blind. Handbook of Clinical Neurology.

[21] Ungur A.P., Bârsan M., Socaciu A.I., Râjnoveanu A.G., Ionuț R., Goia L., Procopciuc L.M. (2024). A narrative review of burnout syndrome in medical personnel. Diagnostics.

[22] Zhu H., Zhou Z., Xu Y., Chen J., Lin D., Li S., Chen X. (2025). Analysing the effect of social jetlag on burnout among shift nurse using a chained mediation model. Sci. Rep..

[23] Czyż-Szypenbejl K., Mędrzycka-Dąbrowska W. (2024). The Impact of Night Work on the Sleep and Health of Medical Staff-A Review of the Latest Scientific Reports. J. Clin. Med..

[24] Saintila J., Soriano-Moreno A.N., Ramos-Vera C., Oblitas-Guerrero S.M., Calizaya-Milla Y.E. (2024). Association between sleep duration and burnout in healthcare professionals: A cross-sectional survey. Front. Public Health.

[25] Begin A.S., Hata S., Berkowitz L.R., Plessow F., Lawson E.A., Emptage N., Armstrong K. (2022). Biomarkers of Clinician Burnout. J. Gen. Intern. Med..

[26] Młynarska A., Bronder M., Kolarczyk E., Manulik S., Młynarski R. (2022). Determinants of Sleep Disorders and Occupational Burnout among Nurses: A Cross-Sectional Study. Int. J. Environ. Res. Public Health.

[27] Purvanova R.K., Muros J.P. (2010). Gender differences in burnout: A meta-analysis. J. Vocat. Behav..

[28] Li L.Z., Yang P., Singer S.J., Pfeffer J., Mathur M.B., Shanafelt T. (2024). Nurse Burnout and Patient Safety, Satisfaction, and Quality of Care. JAMA Netw. Open.

[29] Alimoglu M.K., Donmez L. (2005). Daylight exposure and other predictors of job burnout among nurses. J. Psychosoc. Nurs. Ment. Health Serv..

