# Supplementary material for: Melatonin and Cortisol Suppression and Circadian Rhythm Disruption in Burnout Among Healthcare Professionals: A Systematic Review
Source: Clin Pract. 2025 Oct 29;15(11):199. doi: 10.3390/clinpract15110199 (PMC12651070; doi:10.3390/clinpract15110199)
Supplement: Supplementary file 1 [file clinpract-15-00199-s001.zip › clinpract-3897944-supplementary.pdf]

PRISMA 2020 Checklist

Section and Topic	Item #	Checklist item	Location where item is reported
**TITLE : Melatonin Dysregulation and Circadian Disruption in Healthcare Professionals- A Systematic Review**			
Title	1	Identifies the report as a systematic review.	1st page -Title page (“Melatonin Dysregulation and Circadian Disruption in Healthcare Professionals—A Systematic Review”)
**ABSTRACT**			
Abstract	2	Structured abstract with background, objectives, methods (databases, timeframe, eligibility), results, and conclusions.	Abstract, p.1
**INTRODUCTION**			
Rationale	3	Explains burnout as an occupational hazard and its biological mechanisms (HPA axis, circadian misalignment).	Introduction, p.1–2.
Objectives	4	States aim: to synthesize evidence on burnout, melatonin, and circadian disruption in healthcare workers and assess translational potential.	End of Introduction, p.2.
**METHODS**			
Eligibility criteria	5	Inclusion: English, 2013–2025, healthcare professionals, validated burnout tools (MBI, CBI), melatonin or circadian measures; Exclusion: animal studies, no burnout/circadian outcomes, non-peer-reviewed.	Section 2.2, p.3
Information sources	6	Databases: PubMed, Scopus, Web of Science, PsycINFO; manual reference screening; last search March 2025.	Section 2.3, p.3.
Search strategy	7	Boolean search strings using MeSH and free-text terms; adapted per database.	Section 2.3, p.3.
Selection process	8	Two independent reviewers screened titles/abstracts and full texts; disagreements resolved by discussion/third reviewer.	Section 2.4, p.4
Data collection process	9	Pre-designed piloted form; two reviewers independently extracted data (authors, country, design, burnout tool, melatonin/circadian measures).	Section 2.4, p.4.
Data items	10a	Burnout scores, melatonin levels (serum/saliva/urine), DLMO, chronotype, cortisol, sleep measures.	Section 2.2–2.4, p.3–4.
	10b	Demographics (age, sex, BMI, specialty, shift work), study design, intervention type.	Section 2.4, p.4.
Study risk of bias assessment	11	Newcastle-Ottawa Scale (NOS); assessed independently by two reviewers.	Section 2.5, p.4.
Effect measures	12	Correlations, regression coefficients, and descriptive measures; narrative synthesis for heterogeneous studies.	Section 2.6, p.4.
Synthesis methods	13a<br>13b	Narrative synthesis grouped into three domains (burnout-melatonin link, chronotype/shift influence, intervention outcomes). Subgroup analysis of night-shift workers. No meta-analysis due to heterogeneity.	Section 2.6, p.4.

PRISMA 2020 Checklist

Section and Topic	Item #	Checklist item	Location where item is reported
	13c		
	13d		
	13e		
	13f		
Reporting bias assessment	14	Not formally performed; noted as a limitation.	Discussion, p.10
Certainty assessment	15	Not conducted; acknowledged as a limitation.	Discussion, p.10.
**RESULTS**			
Study selection	16a	PRISMA flow diagram (Figure 1); 1,134 records → 15 studies included.	Section 3, p.5 & Figure 1.
	16b	Exclusions (e.g., no burnout or melatonin measures, animal studies, low quality) explained.	Section 3, p.5.
Study characteristics	17	Table 1 summarizes country, design, sample, burnout tool, melatonin measure, and findings.	Table 1, p.6–7.
Risk of bias in studies	18	Assessed with NOS and described narratively.	Section 2.5, p.4.
Results of individual studies	19	Summarized in Table 1 (key findings per study).	Results, Table 1, p.6–7.
Results of syntheses	20a	Grouped by theme (melatonin-burnout association, chronotype/shift work influence, interventions). Subgroup analysis of 26 night-shift participants (burnout, melatonin, cortisol).	Section 3, p.5–8
	20b		
	20c		
	20d		
Reporting biases	21	No formal bias assessment; acknowledged as limitation.	Discussion, p.10.
Certainty of evidence	22	Not conducted; limitation noted.	Discussion, p.10.
**DISCUSSION**			
Discussion	23a	Explains biological links between burnout and circadian dysregulation (melatonin, cortisol, HPA axis) and relevance of interventions.	Section 4, p.8–10.
	23b	Cross-sectional designs, heterogeneity of tools, inconsistent sampling times.	Discussion, p.10.
	23c	English-only studies, no formal publication bias or GRADE assessment.	Discussion, p.10.
	23d	Highlights potential for melatonin as a biomarker, chronotherapeutic interventions, and occupational health applications.	Conclusions, p.11–12.
**OTHER INFORMATION**			

PRISMA

PRISMA 2020 Checklist

Section and Topic	Item #	Checklist item	Location where item is reported
Registration and protocol	24a	PROSPERO registration (CRD420251045486); protocol referenced; no amendments reported.	Section 2.1, p.3.
	24b		
	24c		
Support	25	No external funding.	Funding statement, p.13.
Competing interests	26	None declared.	Conflicts of Interest, p.13.
Availability of data, code and other materials	27	Dataset available upon request.	Data Availability, p.13.

From: Page MJ, McKenzie JE, Bossuyt PM, Boutron I, Hoffmann TC, Mulrow CD, et al. The PRISMA 2020 statement: an updated guideline for reporting systematic reviews. BMJ 2021;372:n71. doi: 10.1136/bmj.n71.
This work is licensed under CC BY 4.0. To view a copy of this license, visit https://creativecommons.org/licenses/by/4.0/
